# The Flagellar Regulon of *Legionella—*A Review

**DOI:** 10.3389/fcimb.2017.00454

**Published:** 2017-10-20

**Authors:** Sandra Appelt, Klaus Heuner

**Affiliations:** ^1^Highly Pathogenic Microorganisms, Centre for Biological Threats and Special Pathogens, Robert Koch Institute, Berlin, Germany; ^2^Cellular Interactions of Bacterial Pathogens, Centre for Biological Threats and Special Pathogens, Robert Koch Institute, Berlin, Germany

**Keywords:** *Legionella*, flagellar regulon, flagellin, virulence, FliA, FleQ, alternative sigma factor

## Abstract

The *Legionella* genus comprises more than 60 species. In particular, *Legionella pneumophila* is known to cause severe illnesses in humans. Legionellaceae are ubiquitous inhabitants of aquatic environments. Some Legionellaceae are motile and their motility is important to move around in habitats. Motility can be considered as a potential virulence factor as already shown for various human pathogens. The genes of the flagellar system, regulator and structural genes, are structured in hierarchical levels described as the flagellar regulon. Their expression is modulated by various environmental factors. For *L. pneumophila* it was shown that the expression of genes of the flagellar regulon is modulated by the actual growth phase and temperature. Especially, flagellated *Legionella* are known to express genes during the transmissive phase of growth that are involved in the expression of virulence traits. It has been demonstrated that the alternative sigma-28 factor is part of the link between virulence expression and motility. In the following review, the structure of the flagellar regulon of *L. pneumophila* is discussed and compared to other flagellar systems of different *Legionella* species. Recently, it has been described that *Legionella micdadei* and *Legionella fallonii* contain a second putative partial flagellar system. Hence, the report will focus on flagellated and non-flagellated *Legionella* strains, phylogenetic relationships, the role and function of the alternative sigma factor (FliA) and its anti-sigma-28 factor (FlgM).

## Introduction and overview

The Legionellaceae family consists of a single genus: *Legionella* that comprises more than 60 species so far (Gomez-Valero et al., 2009; Bajrai et al., 2016; Khodr et al., 2016). New species are identified continuously (i.e., *Legionella drancourtii, Legionella gresilensis*, and *Legionella beliardensis*), extending the list of known *Legionella* species (Lo Presti et al., 2001; La Scola et al., 2004; Gomez-Valero et al., 2009; Rizzardi et al., 2015; Bajrai et al., 2016; Khodr et al., 2016). More than 20 pathogenic *Legionella* species are known today that differ in their ability to infect hosts and to cause severe to mild diseases in humans (Rizzardi et al., 2015). Human pathogens known to cause the Legionnaires' disease—an atypical pneumonia—are for instance *Legionella pneumophila, Legionella micdadei*, and *Legionella longbeachae* (Yu et al., 2002; Whiley and Bentham, 2011). *Legionella* known to cause the Pontiac fever—a mild flu-like disease—are for instance *Legionella feelei, L. micdadei* and *Legionella anisa*, but also *L. pneumophila*. Often *Legionella* strains of the same species and same serogroup cause one of the mentioned diseases (Swanson and Hammer, 2000; Fields et al., 2002; Wang et al., 2015).

Nevertheless, it was assumed that humans are accidental hosts of *Legionella* species within which the bacterium replicates (Horwitz and Silverstein, 1980; Cianciotto et al., 1989; Horwitz, 1992; Fields, 1996; Neumeister et al., 1997; Newton et al., 2010). Known natural hosts are protozoa, especially free-living amoebae: *Acanthamoeba* spp., *Naegleria* spp., or *Hartmanella vermiformis* (Barbaree et al., 1986; Rowbotham, 1986; Fields, 1996; Atlas, 1999; Fields et al., 2002; Greub and Raoult, 2004; Abdel-Nour et al., 2013; Richards et al., 2013; Cateau et al., 2014). Accordingly, in general, *Legionella* species are prevalent inhabitants of soil, mud and above all of aquatic environments (Fliermans et al., 1981; Fields, 1996; Atlas, 1999; Gomez-Valero et al., 2009; Declerck, 2010; Schalk et al., 2014; Currie and Beattie, 2015). The ability of *L. pneumophila* to grow within biofilms made by *Klebsiella pneumoniae* or *Pseudomonas aeruginosa* in aquatic or wet environments raised questions about their host-free persistence (Stewart et al., 2012). In connection with favorable aquatic habitats and potential protozoa hosts, especially flagella-driven motility of some *Legionella* spp. is an important feature needed to move around, to find new hosts and to form maybe even biofilms (Kirov et al., 2004; Danhorn and Fuqua, 2007; Heuner and Albert-Weissenberger, 2008). For *Legionella* infecting humans, motility may also be crucial for spreading within lungs of patients, as flagellated forms of *L. pneumophila* were detected in alveolar spaces (Chandler et al., 1980; Jager et al., 2014). Recently, it was published that *L. feelei* strains that cause Legionnaires' disease are flagellated while *L. feelei* strains that cause the Pontiac fever are non-flagellated (Wang et al., 2015). The majority of *Legionella* species are flagellated (Elliott and Johnson, 1981; Bornstein et al., 1991; Bangsborg et al., 1995; Heuner et al., 1995), but not all pathogenic *Legionella* have a complete flagellar regulon (i.e., *L. longbeachae* and *Legionella oakridgensis*, see below) (Orrison et al., 1983; Heuner et al., 1995; Cazalet et al., 2010; Kozak et al., 2010; Brzuszkiewicz et al., 2013).

The assumption that the expression of flagella and virulence are linked was already made at an early stage (Rowbotham, 1986) and later on confirmed. It was shown that there is a regulatory link between the expression of a virulent phenotype and the flagellum (Pruckler et al., 1995; Byrne and Swanson, 1998; Hammer et al., 2002; Gal-Mor and Segal, 2003; Molofsky et al., 2005; Heuner and Albert-Weissenberger, 2008; Albert-Weissenberger et al., 2010; Schulz et al., 2012). The expression of flagellar genes is regulated on the flagellar regulon, extensively investigated in *L. pneumophila* due to its biphasic intracellular life cycle during which the bacterium undergoes a shape change. Within the host, after replication inside of *Legionella*-containing vacuoles (LCVs), when nutrients become limited, *L. pneumophila* differentiates into a flagellated, non-replicating form. The flagellated, transmissible, mature form is stress-resistant, virulent and metabolically resting as well as infectious (abbr. MIF) (Rowbotham, 1986; Byrne and Swanson, 1998; Heuner et al., 1999; Swanson and Hammer, 2000; Faulkner and Garduno, 2002; Garduno et al., 2002; Hammer et al., 2002; Molofsky and Swanson, 2004; Fonseca and Swanson, 2014; Eisenreich and Heuner, 2016). The actual release process of mature forms is still under discussion: either the bacteria are released from the LCV into the environment by lysis of the host or the bacteria are released first into the cytosol of the host and then after an additional putative round of replication into the environment (Rowbotham, 1986; Molmeret et al., 2004). The latter hypothesis implies that the flagellum is produced inside the cytosol of the host and not in LCVs as proposed earlier. Furthermore, there is also a possibility that the bacteria are released by the host *via* a non-lytic mechanism (Chen et al., 2004; Bouyer et al., 2007; Berk et al., 2008). However, the released form is well-prepared to reinfect new hosts or to differentiate into a viable-but-nonculturable form (VBNC) meant to enable a long-term survival of the bacteria (Rowbotham, 1986; Steinert et al., 1997; Ohno et al., 2003; Molmeret et al., 2010; Al-Bana et al., 2014). VBNC forms can be resuscitated when they are taken up by amoebae (Steinert et al., 1997; Ohno et al., 2003; Al-Bana et al., 2014). Further, different morphological forms of *L. pneumophila* have been recently discussed (Robertson et al., 2014). Next to the biphasic intracellular life cycle it was shown that *L. pneumophila* exhibits also a life stage-specific bipartite metabolism, an area for further investigations (Schunder et al., 2014; Eisenreich and Heuner, 2016; Gillmaier et al., 2016; Hauslein et al., 2016).

## Motility of *Legionella*

Different forms of bacterial motility are known including swarming, twitching and sliding. Notably the flagellum—next to pili—allows bacteria to move. Bacterial motility is often related to chemotactic behavior that enables a bacterium to locate special environmental conditions and to get closer to higher concentrations of attractants (Szurmant and Ordal, 2004; Hazelbauer et al., 2008; Micali and Endres, 2016). Some *Legionella* have a chemotaxis system (*L. longbeachae, Legionella parisiensis*, and *Legionella bozemanii*) but most *Legionella* do not have the corresponding genes (e.g., *L. pneumophila, L. micdadei*, and *L. oakridgensis*). Moreover, the ability of *Legionella* to swarm and to show a chemotaxis behavior has not yet been reported.

Twitching motility is based on a functional type IV pilus. The ability to move forward by twitching has been reported for *L. pneumophila* (Coil and Anne, 2009; Hoppe et al., 2017). In addition, sliding motility, a surfactant-mediated motility, has been described for *L. pneumophila* (Stewart et al., 2009).

## The flagellum and the flagellar regulon

### The structure of the flagellum and flagellar systems

**Figure 1:**
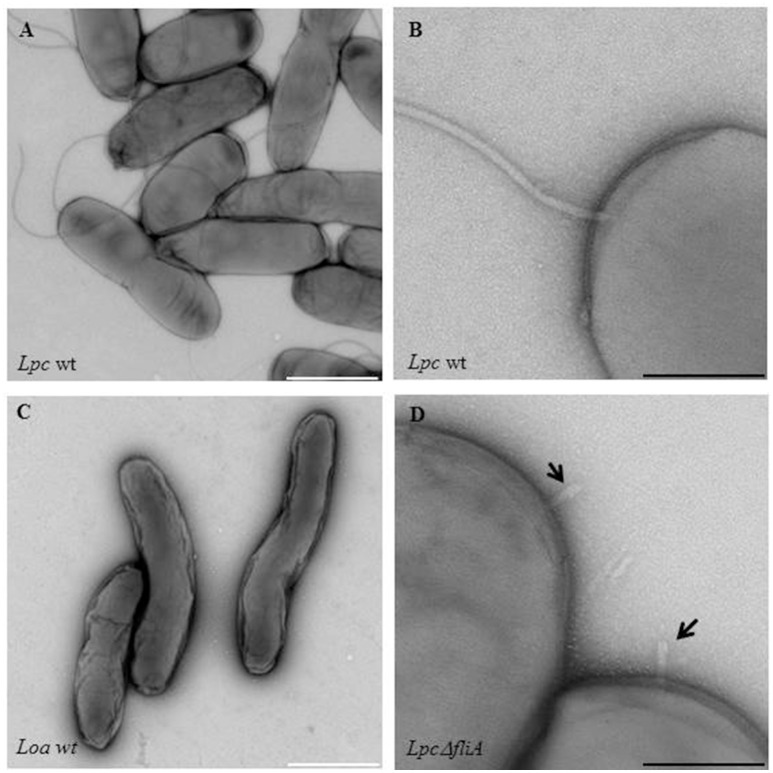
Electron microscopic images of flagellated and non-flagellated *Legionella* wild-type strains as a flagella mutant strain. Shown are **(A,B)** *Legionella pneumophila* Corby (Lpc wt) and **(C)** *Legionella oakridgensis* (Loa wt) wild-type strains that are either representative of flagellated or non-flagellated *Legionella*. An electron microscopic image of a *fliA L. pneumophila* mutant strain (Lpc Δ*fliA*), is also shown **(D)**. The straight hook structure of the mutant strain is indicated by black arrows **(D)**. Bacteria were grown in AYE medium at 30°C and the samples were stained with 0.5% uranyl acetate. White scale bars: 1 μm; black scale bars: 200 nm.

**Figure 2:**
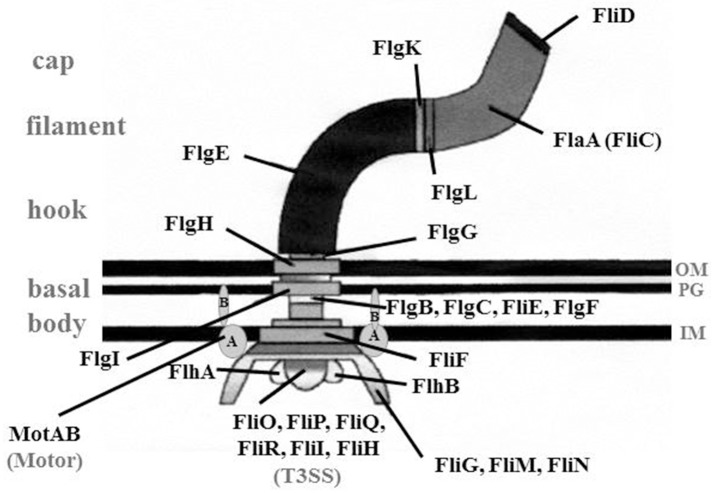
Overview of the structure of a flagellum. Schematic drawing of a flagellum consisting of basal body, hook structure and filament. Cap, FliD protein that is essential for the assembly of the filament, Motor, MotAB proteins that provide energy for the rotation; T3SS, type III-like secretion system; FlgD (not shown), the hook cap protein needed to incorporate FlgE; IM, inner membrane; OM, outer membrane; PG, peptidoglycan layer; (Heuner and Albert-Weissenberger (2008), modified).

Most *Legionella* species are motile due to a single polar flagellum (Figure 1) (Chandler et al., 1980; Elliott and Johnson, 1982; Heuner et al., 1995). More than 50 genes are involved in the expression of functional flagella, and due to high metabolic costs, a tight regulation is essential (Chilcott and Hughes, 2000; McCarter, 2006; Osterman et al., 2015). The flagellum of *Legionella* consists of a basal body, a hook structure and a filament (Figure 2; Heuner and Steinert, 2003; Heuner and Albert-Weissenberger, 2008). For the assembly of the flagellum, needed proteins (hook, rod and the filament forming proteins) are exported out of the cell by a flagellum-specific export apparatus, a type III-like secretion system (T3SS) (Heuner and Albert-Weissenberger, 2008; Altegoer and Bange, 2015).

The basal body consists of a rod, three rings [“membrane/supramembrane” (MS), “peptidoglycan” (P), and “lipopolysaccharide” (L)] and a motor switch complex (MotAB). The MotAB is providing the energy for the rotation of the flagellum (Minamino and Imada, 2015). For the formation of the slightly curved hook structure, FlgE and FlgD are essential. The hook cap protein FlgD assists when FlgE is incorporated into the hook structure (Altegoer and Bange, 2015).

Interestingly, an uncommonly straight hook has been reported for *L. pneumophila* mutant strains (Δ*fliA*, Δ*fliD*, and Δ*flaA*, i.e., a flagellin mutant) (Figure 1) (Schulz et al., 2012). The findings give credit to the assumption that *L. pneumophila* might have a straight hook that is hard to detect in wildtype strains. The filament consists mainly out of a single protein, the flagellin (FlaA or FliC) (Figure 2). The cap protein FliD is essential for the assembly of flagellin subunits into the filament. To assemble the filament, flagellin is exported through the filament structure by a flagellum-specific export apparatus (T3SS) and assembled at the tip of the filament (Heuner and Albert-Weissenberger, 2008; Altegoer and Bange, 2015).

More details about the flagellum structure can be found in dedicated review articles (Aldridge and Hughes, 2002; Heuner and Steinert, 2003; Macnab, 2003; Pallen et al., 2005; Heuner and Albert-Weissenberger, 2008; Altegoer and Bange, 2015).

**Figure 3:**
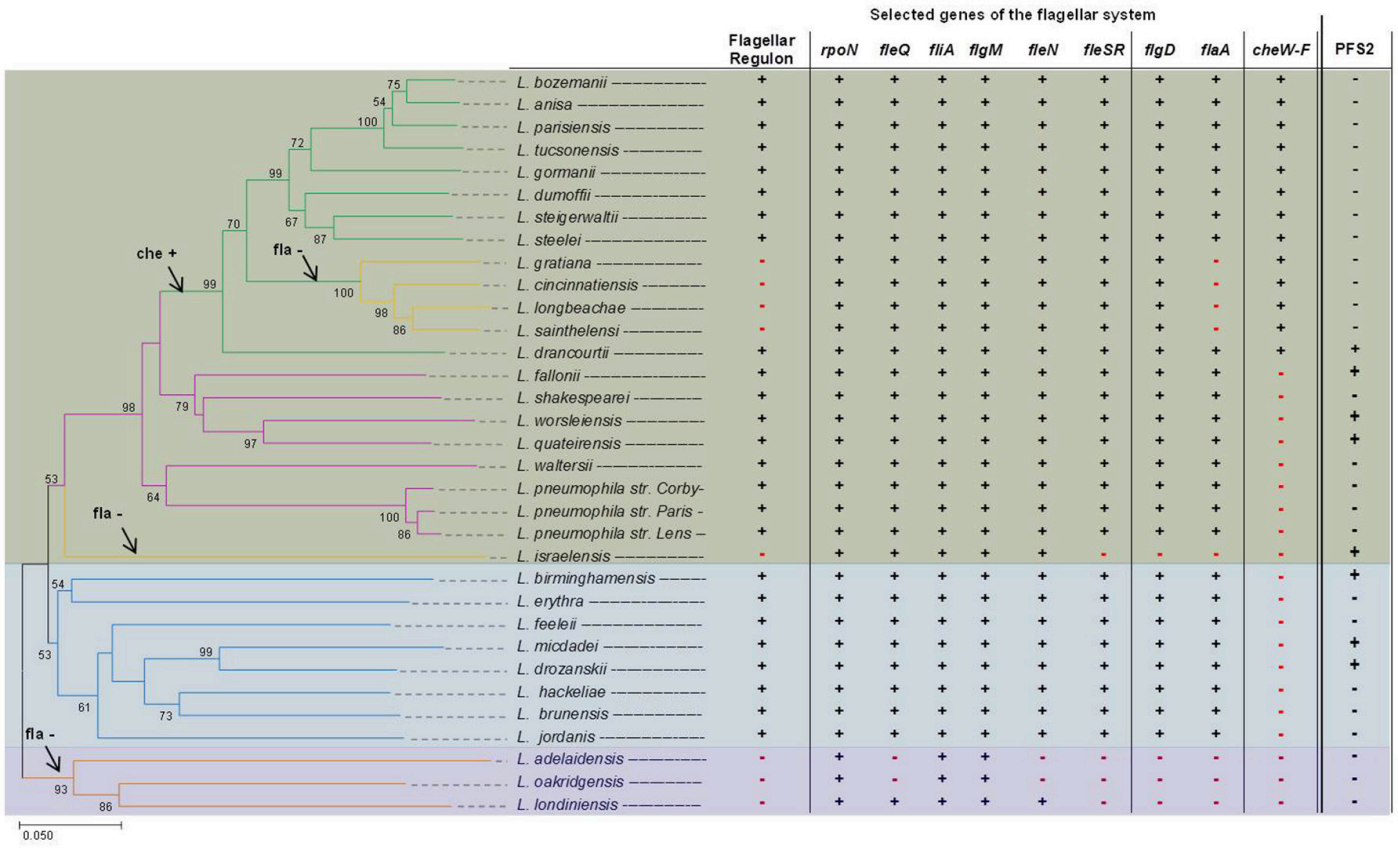
Evolutionary relationships of *Legionella* species and distribution of flagellar regulon and chemotaxis genes. Shown is a phylogenetic tree of *Legionella* spp. based on the *fliA* gene (left side) and also the distribution of flagellar regulon and chemotaxis genes (right side). The phylogenetic tree was reconstructed on the basis of the *fliA* gene using the Neighbor-Joining method (Saitou and Nei, 1987) and a bootstrap of 1,000 (Felsenstein, 1985). The bootstrap >50 is reported next to the branches. The phylogenetic distances were computed using the Tamura-Kumar method (Tamura and Kumar, 2002) and refers to the units of the number of base substitutions per site. For the evolutional analysis, performed in MEGA7 (Kumar et al., 2016), position containing gaps and/or missing data were eliminated. To determine whether selected genes belonging to the flagellar regulon and chemotaxis genes are present among investigated *Legionella*, an *in silico* BLAST search was performed using *Legionella* draft genomes recently published by Burstein et al. (2016). The complete structure of genes of the flagellar regulon of *L. pneumophila* strain Corby, *L. longbeachae, L. israeliensis*, and *L. oakridgensis* are given in Figure 5. Data for *L. pneumophila* strains were taken from Cazalet et al. (2010), Glockner et al. (2008), and Albert-Weissenberger et al. (2010). *Legionella* clade I is highlighted in green, clade II in blue and clade III in purple. On the left, in the phylogenetic tree, possible time points when the flagellin gene (*flaA*) and the chemotaxis (*che*) genes were lost (–) or acquired (+) are indicated with arrows. On the right, the presence (+) and/or absence (–) of selected genes belonging to the flagellar regulon, the chemotaxis operon or an operon encoding a putative second flagellum or a putative T3SS (PFS2, *based on unpublished data*) are indicated, respectively.

Next to the regular flagellar system, a second putative flagellar system was suspected for two *Legionella* species: *Legionella fallonii* and *L. micdadei* (Gomez-Valero et al., 2014). Comparative genome analysis led to the suspicion that the strains do have homologs to flagellar genes of *L. pneumophila*. The identified genetic region is comprised of genes that encode a putative basal body, a secretion system, as well as a putative hook structure. No homologs to *flaA* or *fliD* were found in the predicted genomic region. Yet, further investigations are needed to find out if a T3SS or a putative second flagellum is encoded. Additionally, *in silico* investigation, performed on the draft genome sequence of *Legionella israelensis*, identified a similar operon. A BLAST search using the operon (10,041 bp, ctg_064, *L. israeliensis* draft-genome; Burstein et al., 2016) as query identified similar genes in *L. drancourtii, L. fallonii, L. worsleiensis, L. quateirensis, L. birminghamensis*, and *L. drozanskii* (Heuner, unpublished results). Initial findings show that the operon is present in two out of three *Legionella* clades (Figure 3), leaving a margin for additional studies.

### The flagellar regulon

**Figure 4:**
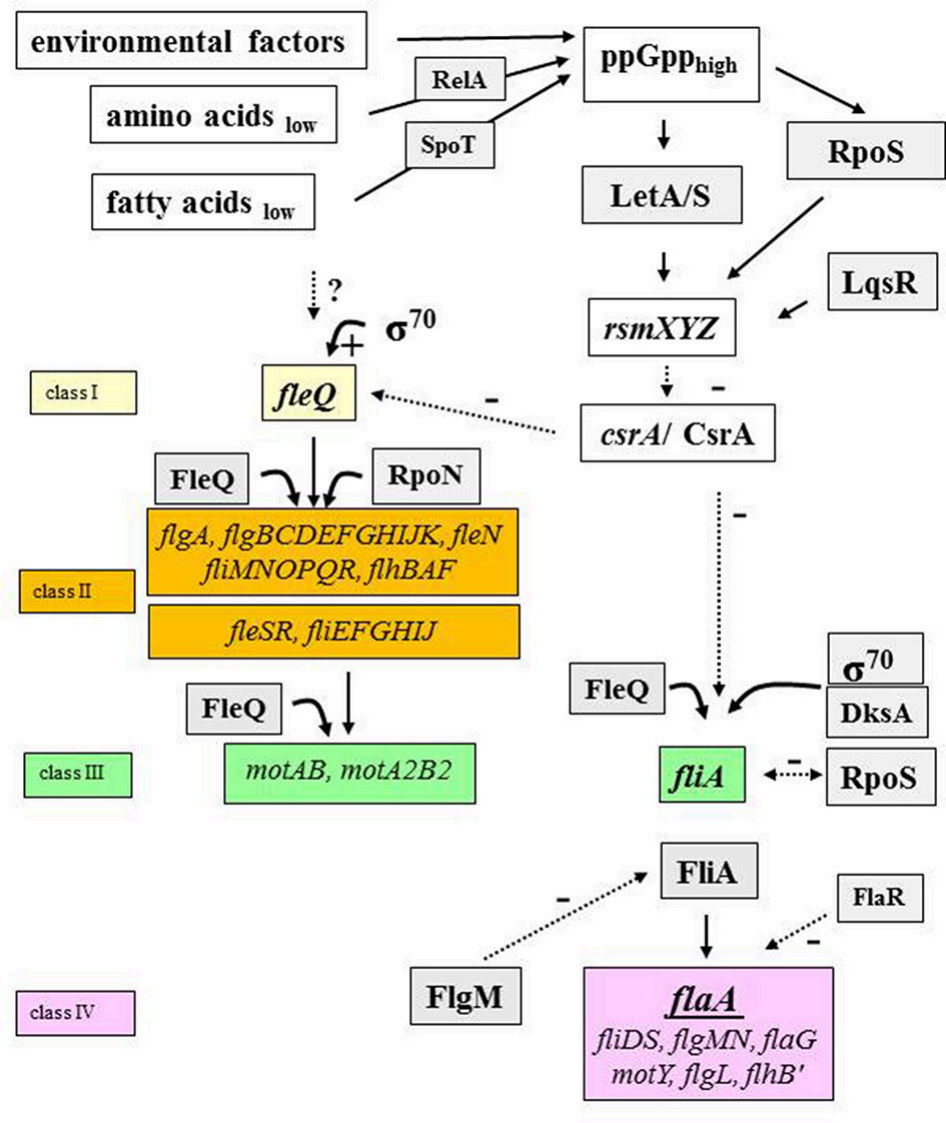
The flagellar regulon cascade. The regulatory cascade which leads to the expression of proteins that are needed to construct a functional flagellum is shown (Heuner and Albert-Weissenberger, 2008; Sahr et al., 2017, modified). Specific environmental factors lead to the activation of the pathway through alarmone (ppGpp) accumulation. Some main players of the flagellar regulon cascade are: the activator protein FleQ, the alternative sigma factor 28 (FliA), the two-component system (LetA/S) and the carbon storage regulator (CsrA) as examples. FleQ is the master regulator of the flagellar genes (Class II and class III). FliA is the regulator of flagellar genes of class IV. Black lined, continuous arrows refer to positive regulation events. The broken lines in black refer to negative regulatory effects of respective factors. Question marks (?) indicate pathway points with putative mode of actions that need to be specified by further investigations. RsmXYZ, regulatory RNAs; FlaA, flagellin; FlaR, transcriptional regulator (LysR family); FleQ and FleR, sigma factor 54 activator protein; FlgM, anti-sigma 28 factor; RpoS, alternative sigma 38 factor.

The expression of flagellar genes of *L. pneumophila* is regulated in a hierarchical cascade (Figure 4) (Heuner et al., 1995, 2006; Heuner and Steinert, 2003; Jacobi et al., 2004; Albert-Weissenberger et al., 2010; Schulz et al., 2012). Their expression depends on growth phase, temperature, medium viscosity and nutrient availability (e.g., amino acids and fatty acids) (Ott et al., 1991; Byrne and Swanson, 1998; Heuner et al., 1999, 2006; Heuner and Albert-Weissenberger, 2008).

In short, intracellular alarmone accumulation—ppGpp, a signal molecule, produced when environmental conditions are unfavorable (e.g., limited nutrient supply)—is regulated by RelA and SpoT. RelA senses the amount of available intracellular amino acids and SpoT senses the amount of intracellular fatty acids (Hammer and Swanson, 1999; Dalebroux et al., 2009, 2010).

Alarmone accumulation triggers the activation of an alternative stationary-phase sigma factor (RpoS) and of the two component system LetA/S (Byrne and Swanson, 1998; Hammer et al., 2002; Zusman et al., 2002; Molofsky and Swanson, 2004; Dalebroux et al., 2009, 2010; Edwards et al., 2009; Rasis and Segal, 2009; Sahr et al., 2009) (Figure 4). RpoS and LetA/S promote the transcription of small regulatory RNAs (RsmX, RsmY, RsmZ). RsmX plays a role in the virulence of *L. pneumophila* (Sahr et al., 2012). The transcription of *rsmZ*/*rmsY* is also influenced by a quorum sensing system regulator, LqsR (Tiaden et al., 2007; Schell et al., 2016). The two regulatory RNAs are able to bind a number of carbon storage regulator molecules (CsrA) at once (Sahr et al., 2017). CsrA is a negative regulator and through the binding on RsmY or RsmZ, other targets of the regulatory RNAs can be expressed. The expression of transmissive traits starts and main activator proteins (e.g., FleQ) are produced and flagellar genes are expressed (Zusman et al., 2002; Molofsky and Swanson, 2003; Rasis and Segal, 2009; Sahr et al., 2009, 2017; Albert-Weissenberger et al., 2010). Notably, some parts of the function of the negative regulator CsrA (*flaA* expression and motility of *L. pneumophila*) can be “complemented” by ectopically expressed *csrT*, a CsrA-like regulatory gene associated with integrative conjugative elements (Abbott et al., 2015).

CrsA is also controlling a major regulator involved in the expression of flagella, FleQ (Sahr et al., 2017). FleQ is responsible for the expression of early flagellar genes belonging to class II and III genes in an RpoN-dependent and RpoN-independent pathway (Jacobi et al., 2004; Albert-Weissenberger et al., 2010; Schulz et al., 2012). RpoN is an enhancer-binding protein encoding an alternative sigma factor that initiates transcription when activator proteins like FleQ, FleR, and PilR (Jacobi et al., 2004) are present. When class II and III genes are expressed, the activity of FliA—the alternative sigma factor—leads to the expression of class IV genes and the assembly of the flagellum (Figure 4). RpoN and FleR seem to be responsible for a negative feedback loop on flagellar genes (Albert-Weissenberger et al., 2010). It was found that RpoS and FlaR (transcriptional regulator FlaR, LysR family member) are also involved in the expression of the flagellin gene (Heuner et al., 2000; Bachman and Swanson, 2001, 2004; Rasis and Segal, 2009; Sahr et al., 2009). The production of FlaA is also regulated by cyclic di-GMP, shown by the analysis of a gene (*cdgS13*) coding for a protein with diguanylate cyclase activity (Levi et al., 2011). The influence of cyclic di-GMP on flagellum-based motility has been shown for other bacteria than *Legionella* species (Wolfe and Visick, 2008).

## The flagellum and virulence

Already early on, it has been hypothesized that virulence and flagellum expression are genetically linked with each other. It has been shown that motile, transmissive *Legionella* were more infectious for amoebae than non-motile replicative phase *Legionella* (Rowbotham, 1986; Pruckler et al., 1995; Bosshardt et al., 1997; Byrne and Swanson, 1998; Hammer et al., 2002; Heuner et al., 2002; Molofsky et al., 2005; Heuner and Albert-Weissenberger, 2008). Different experiments could show that the motility but not the flagellin promotes the contact with host cells. Motility increases the infectivity and the fitness. Furthermore, it turned out that the flagellum is not necessary for intracellular replication (Pruckler et al., 1995; Dietrich et al., 2001; Polesky et al., 2001; Heuner et al., 2002; Jacobi et al., 2004; Molofsky et al., 2005; Schulz et al., 2012).

The four major regulators of the flagellar regulon (RpoN, FleQ, FleSR, FliA) seems to be involved in the invasion process of *L. pneumophila* into hosts. These findings point out the proposed link between virulence traits and flagellum expression (Dietrich et al., 2001; Hammer et al., 2002; Molofsky et al., 2005; Heuner and Albert-Weissenberger, 2008; Albert-Weissenberger et al., 2010; Schulz et al., 2012). Especially the FliA regulon plays an important role (please, see the section: FliA and its implication in virulence below). *FliA*, but not the flagellin (*flaA)*, is involved into the ability of *L. pneumophila* to form biofilm that allow bacteria to survive whenever environmental conditions are not favorable. (Mampel et al., 2006). Notably unwanted biofilms are a health issue causing a significant amount of nosocomial infections (Bryers, 2008). *Legionella* are known to survive within biofilm of other bacteria (e.g., *Klebsiella pneumophila* and *Pseudomonas aeruginosa*) (Molofsky et al., 2005; Stewart et al., 2012). More recently, findings about *L. pneumophila's* ability to form biofilms by itself in natural environments and on medical devices have attracted attention (Lau and Ashbolt, 2009; Abu Khweek et al., 2013). Biofilm formation is regulated by temperature, surface material and intracellular growth (Konishi et al., 2006; Piao et al., 2006; Bigot et al., 2013) and biofilm-derived *L. pneumophila* do not express flagellin (Abu Khweek et al., 2013). More information about biofilms and *L. pneumophila* can be found in a recent review (Abdel-Nour et al., 2013).

The flagellum also affects the resistance of hosts to Legionnaires' disease and when *Legionella* do not produce flagellin they can evade the innate immune response in macrophages (Hawn et al., 2003; Molofsky et al., 2006; Ren et al., 2006; Abu Khweek et al., 2013). Resistance is mediated by the Naip5/Ipaf-dependent recognition of flagellin, which induces a protective immunity in non-A/J mouse models (Ricci et al., 2005). Detailed information about addressed points can be found in dedicated reviews (Fontana and Vance, 2011; Schell et al., 2016; Mascarenhas and Zamboni, 2017).

## The alternative sigma factor 28

### FliA expression

One of the major regulators involved in the expression of the flagellum is FliA and an increased alarmone level leads to accumulation of functional FliA (Bruggemann et al., 2006; Heuner et al., 2006; Dalebroux et al., 2010). The alternative sigma factor (σ^28^) is directly involved in the regulation and expression of the flagellin gene (*flaA*) and others (Figure 4). A *fliA* mutant of *L. pneumophila* does not produce flagellin and is consequently non-flagellated. Moreover, a Δ*fliA* mutant of *Escherichia coli* can be completed with a *fliA* gene of *L. pneumophila* (Heuner et al., 1995, 1997, 2002; Bruggemann et al., 2006; Albert-Weissenberger et al., 2010; Schulz et al., 2012).

The expression of flagellar class III and IV genes is induced in a FleQ-dependent manner. The FliA-regulated class IV genes are involved in the assembly of the filament and flagella motility (*flgL, fliD, flaA, motY*). Both lead to the complete synthesis of the flagellum (Jacobi et al., 2004; Albert-Weissenberger et al., 2010). The *fliA* gene itself is expressed in a FleQ-dependent but RpoN-independent manner (Albert-Weissenberger et al., 2010). Nevertheless, FleQ and RpoN are not necessary for a basal expression of *fliA*. For a basal expression, *fliA* is transcribed from a putative sigma-70 promoter element and later, during the exponential phase, the expression of *fliA* is induced in a FleQ-dependent manner (Schulz et al., 2012). Accordingly, it was hypothesized that during the exponential phase the basal *fliA* promotor activity may be mediated by DksA independent of the ppGpp concentration, whereas during the post-exponential phase DksA cooperates with ppGpp to activate *fliA* (Dalebroux et al., 2010). The identification of the transcription start point of *fliA* corroborates the presence of a putative DksA binding site, an A/T rich discriminator site (Schulz et al., 2012).

### FliA and its implication in virulence

FliA is a regulator that is also involved in the expression of putative virulence genes (Bruggemann et al., 2006; Albert-Weissenberger et al., 2010; Tlapak et al., 2017).

Several investigations performed on a *fliA* mutant strain of *L. pneumophila* pointed out that the mutant (at low MOI) is not replicating in host cells anymore (*Dictyostelium discoideum*). The *fliA* mutant seems to be less infectious for macrophages and non-cytotoxic to bone marrow-derived macrophages. Moreover, the mutant has a reduced fitness potential in amoebae (Dietrich et al., 2001; Hammer et al., 2002; Heuner et al., 2002; Jacobi et al., 2004; Molofsky et al., 2005; Heuner and Albert-Weissenberger, 2008; Schulz et al., 2012). Likewise, a *fliA* mutant strain of *L. oakridgensis* showed a reduced fitness in its host (*Acanthamoeba lenticulata*) (Tlapak et al., 2017). In addition, another *L. pneumophila fliA* mutant strain exhibited a reduced ability to form biofilms (Mampel et al., 2006).

FliA is obviously a virulence factor, and target genes of *fliA* were investigated to understand its implication for virulence (Bruggemann et al., 2006; Albert-Weissenberger et al., 2010; Tlapak et al., 2017). Target genes of *fliA* in *L. pneumophila* strains include genes of the flagellar regulon (e.g., *flaA* and *flgM*) and others (e.g., *enhA* and *lvrA*), illustrated in Figure 4 and listed in Table 1 (Bruggemann et al., 2006; Albert-Weissenberger et al., 2010; Schulz et al., 2012). Other identified FliA-dependent genes encode for putative virulence factors corroborating the involvement of FliA in the establishment of *Legionella* infections. Identified putative virulence factors are: *lpp0952, lpp1290*, and *lpp0972*. The first one, *lpp0952*, is coding for a GGDEF/EAL and PAS/PAC domain protein (Bruggemann et al., 2006; Albert-Weissenberger et al., 2010). The *two* remaining genes are homologs of the enhanced entry proteins EnhA. Respective homologs were also found in *L. longbeachae* which is non-flagellated, putatively associated with the flagellar system (Kozak et al., 2010).

**Table 1:** Genes belonging to the FliA regulon (FliA target genes) of *L. pneumophila* Paris (Lpp)^*^.

**Gene name**	**Annotation**	**FC**
§ *lpp1294, flaA*	Flagelline	0.003
*lpp1293, flaG*	Unknown	0.007
§ *lpp0972*	Similar to enhanced entry protein EnhA	0.010
*#§ lpp2282*	Unknown	0.024
*lpp1746, fliA*	Sigma factor 28	0.042
*#§ lpp2998*	Similar to conserved hypothetical protein	0.045
§ *lpp1292, fliD*	Flagellar capping protein	0.045
*lpp0197*	Similar to adenine specific DNA methylase	0.046
*lpp1291, fliS*	Similar to flagellar protein FliS	0.048
*lpp1745, motA*	Flagellar motor protein MotA	0.052
§ *lpp1290*	Similar to enhanced entry protein EnhA	0.053
§ *lpp1841*	Unknown	0.059
*lpp0968, flgN*	Hook-associated protein	0.068
*#lpp0969, flgM*	Anti-sigma-28 factor	0.081
*lpp0198*	Similar to Type III RM enzyme- helicase subunit	0.097
*lpp1050*	Unknown	0.114
§ *lpp3034, motY*	Similar to sodium-type flagellar protein MotY	0.116
*lpp1743*	Similar to hypothetical poteins	0.122
§ *lpp0952*	Regulatory protein (GGDEF and EAL domains)	0.122
*lpp2281*	Similar to membrane-associated metalloprotease proteins	0.133
*lpp2376*	Similar to *Legionella vir* region protein LvrA	0.165
*lpp0763*	Weakly similar to *L. pneumophila* IcmL protein	0.187
*lpp1941*	Unknown	0.202
*lpp2634*	Similar to hypothetical proteins	0.228
*lpp1568, plaB*	Phospholipase	0.232
§ *lpp2260*	Unknown	0.262
*lpp2635, flhB′*	Similar to FlhB protein	0.295
*lpp0010*	Similar to GTP-binding protein HflX	0.296
*lpp0009*	Similar to host factor-1 protein	0.366
*plpp0131*	Similar to alanyl tRNA synthetase	0.370
*lpp1742, prfB*	Highly similar to peptide chain release factor 2	0.371
*lpp2386*	Unknown	0.374
*lpp1234, flgL*	Flagellar hook-associated protein FlgL	0.379

* *From Table S7 (Albert-Weissenberger et al., 2010), modified; ^#^Homolog gene belonging to the FliA-regulon of L. oakridgensis (data from Tlapak et al., 2017); ^§^Belonging to the FliA regulon of L. pneumophila Paris replicating in A. castellanii (data from Bruggemann et al., 2006); FC, fold-change values*.

**Figure 5:**
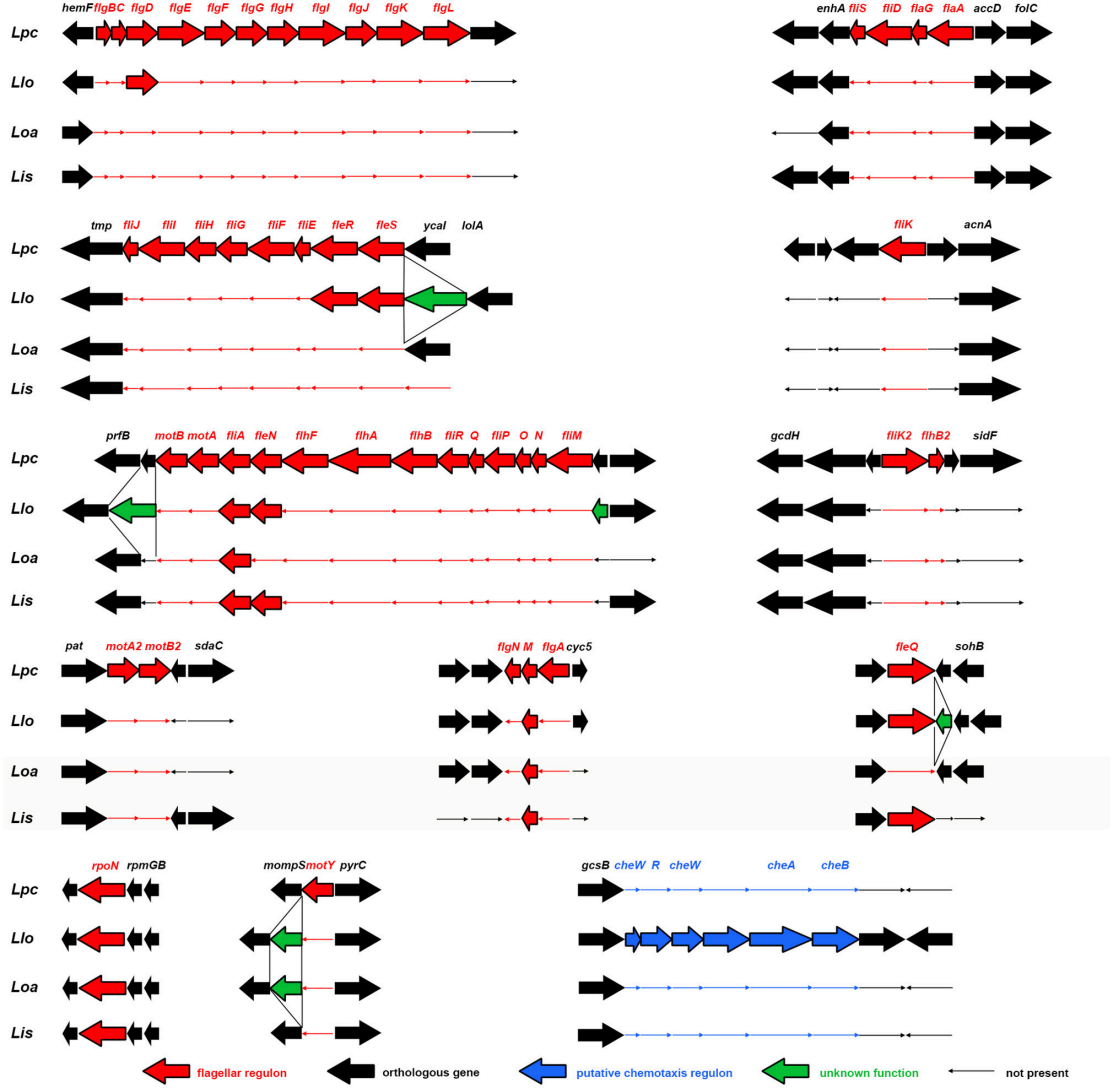
Structure of the operons of the flagellar regulon of *L. pneumophila* Corby (Lpc), *L. longbeachae* (Llo), *L. israeliensis* (Lis), and *L. oakridgensis* (Loa). Genes are indicated by arrows and gene names are given above. Flagellar and chemotaxis genes are indicated in red and blue, respectively; other genes in black. Genes not present in a strain are indicated by thin arrows (Brzuszkiewicz et al., 2013, modified).

Recently, especially *L. oakridgensis* simplified the identification of *fliA* targets potentially involved in virulence (Tlapak et al., 2017). *L. oakridgensis* strains are non-flagellated, the entire flagellar regulon is missing and only homologs of FliA and FlgM are present (Figure 5). *L. oakridgensis* is less infectious then *L. pneumophila*, but still causes Legionnaires' disease. In addition, *L. oakridgensis* replicates in guinea pigs, in human cell lines, in *Acanthamoeba lenticulata* and for growth in media no additional cysteine is needed. (Orrison et al., 1983; Fields et al., 1986; O'Connell et al., 1996; Neumeister et al., 1997; Lo Presti et al., 2001; Brzuszkiewicz et al., 2013). Nevertheless, *L. oakridgensis* exhibits a functional T4SS, homologs of known virulence factors, as well as newly identified virulence factors (Brzuszkiewicz et al., 2013). *L. oakridgensis* is used to investigate FliA since a *fliA L. oakridgensis* knockout will not cause the inactivation of the entire flagellar regulon and target genes of FliA can still be identified as well as genes involved in the expression of virulence traits. However, mutant strain analyses aimed at identifying target genes of FliA in *L. oakridgensis* yielded no results for putative FliA-dependent virulence genes yet (Table 1; Tlapak et al., 2017). Additional investigations are needed including phenotypic characterizations and deletion analyses of further target genes of FliA.

### FliA-FlgM interaction in *Legionella oakridgensis*

Flagellated bacteria regulate the FliA activity often post-transcriptionally. For *Salmonella, Escherichia*, and *Vibrio* species it is known that an anti-sigma-28 factor (FlgM) binds FliA, preventing the binding of FliA to FliA-dependent promoter sites, and FliA-dependent genes are consequently not translated. After assembling of the hook-basal body structure, FlgM is exported and FliA is not repressed anymore (Gillen and Hughes, 1991; Ohnishi et al., 1992; Chilcott and Hughes, 2000; Aldridge et al., 2006). In *Helicobacter pylori*, the FlgM protein is inactivated instead of being exported out of the cells (Rust et al., 2009). FliA-FlgM interactions in *Legionella* are still unknown. Recent findings suggest that FliA-FlgM interaction might be different than in other flagellated bacteria, at least for *L. oakridgensis*. Respective species do not have a flagellum (Figure 1C), a flagellar regulon (Figure 5) and a basal body although *flgM* and *fliA* homologs are present which encode for functional FlgM and FliA proteins (Brzuszkiewicz et al., 2013; Tlapak et al., 2017); consequently, the mechanism controlling FliA-FlgM interactions must be different.

For *L. pneumophila* as well as for *L. oakridgensis* it was found that the expression of FlgM or homologs is sigma-28-dependent (Albert-Weissenberger et al., 2010; Tlapak et al., 2017). Although *L. oakridgensis* has no flagellar system, the expression of *fliA-*dependent genes is growth phase- and temperature-dependent (Heuner et al., 1999; Tlapak et al., 2017). Moreover, for *L. oakridgensis* it was demonstrated that FlgM is a negative regulator of FliA-dependent genes and the protein itself seems to be degraded in a growth phase- and temperature-dependent manner (Tlapak et al., 2017). Thus, it seems likely that, as described for *H. pylori*, FlgM in *L. oakridgensis* is degraded by protease activity instead of being secreted. However, investigations are needed to show if FlgM in *L. pneumophila* is effectively secreted in a basal body-dependent manner.

## Distribution of the flagellar system among *Legionella* species

Phylogenetic reconstruction—based on concatenated amino acid alignment of 78 orthologous ORFs—divided the *Legionella* species into three major clades (clade I and clade II, clade III) (Burstein et al., 2016). Clade I is comprised of most *Legionella* species including *L. pneumophila, L. parisiensis, L. bozemanii*, and *L. longbeachae* (Burstein et al., 2016). Clade II comprises among others *L. feelei* and *L. micdadei* and clade III, a deep-branching clade, includes three members: *Legionella adelaidensis, L. oakridgensis* and *Legionella londiniensis* (Burstein et al., 2016). Phylogenetic reconstructions performed herein, yielded similae results that are given in Figure 3. As opposed to former investigations, the phylogenetic tree was reconstructed on the basis of the *fliA* gene.

The flagellar system can be found in *Legionella* species classified as clade I or II (Cazalet et al., 2004, 2010; Chen et al., 2004; Bruggemann et al., 2006; Glockner et al., 2008; Kozak et al., 2010; Brzuszkiewicz et al., 2013; Gomez-Valero et al., 2014; Burstein et al., 2016), but not in clade III *Legionella*. Clade III *Legionella* do not have a functional flagellar system and do not have most of the flagellar regulon genes, except for *fliA* and its anti-sigma factor *flgM* (*L. oakridgensis, L. adelaidensis*, and *L. londiniensis*) (Cazalet et al., 2010; Brzuszkiewicz et al., 2013; Tlapak et al., 2017) and two additional genes: *fleQ* and *fleN* (*L. londiniensis*) (Figures 3, 5). Also, some clade I *Legionella* species do not have a functional flagellar system (clade I: *L. longbeachae*, Figure 5*, Legionella gratiana, Legionella cincinnatiensis, Legionella sainthelensi*, and *L. israelensis*). It has been hypothesized that the loss of flagellar genes has not happened recently (Kozak et al., 2010). This is corroborated by the finding that *L. longbeachae* and all subclade members are negative for the flagellar regulon but positive for genes coding for the sigma factor FliA, the regulator FleN and the two component system comprising of FleR and FleS, as well as FlgD (Figure 3) (Cazalet et al., 2010; Kozak et al., 2010). Also *L. israelensis* is negative for *flaA* (Heuner et al., 1995) and most flagellar regulon genes, except: *fleQ, fliA, fleN*, and *flgM* (Figure 5). The finding allows to assume that the flagellar system may have been lost at different time points during the evolution of *Legionella* species (Figure 3). In addition, some genes that have regulatory functions outside of the flagellar system are still present (Albert-Weissenberger et al., 2010; Cazalet et al., 2010; Kozak et al., 2010; Tlapak et al., 2017). The same applies to *flgD* which is involved in the assembly of the hook structure of the flagellum with unassigned hypothetical alternative functions.

The investigated *Legionella* genomes were also screened for the presence/absence of genes of major regulators of the flagellar system as well as of the chemotaxis operon (Figure 3). It was found that the genes of the chemotaxis operon are only found in a subclade of the clade I *Legionella*. *L. longbeachae* is the first *Legionella* species described to exhibit chemotaxis genes (Cazalet et al., 2010; Kozak et al., 2010) that do not have flagellar genes. It seemed paradoxical that *L. pneumophila* is flagella positive but chemotaxis negative and *L. longbeachae* is flagella negative but chemotaxis positive. The distribution of the chemotaxis operon may indicate that the chemotaxis operon was acquired by a common 'ancestor' of this sub-tree clade (Figure 3).

## Conclusion

The review aimed to summarize knowledge gained about flagella and the flagellar regulon of different *Legionella* species. The majority of *Legionella* species exhibit genes encoding for a functional flagellum and they are flagellated (Elliott and Johnson, 1981; Bornstein et al., 1991; Bangsborg et al., 1995; Heuner et al., 1995). Motility increases infectivity and fitness, helping the bacteria to reach new hosts after successful replication within protozoan host cells and release into aquatic environments.

Some *Legionella*—including some pathogenic species (e.g., *L. longbeachae*, and *L. oakridgensis*)—are not flagellated and most flagellar regulon genes are absent (Orrison et al., 1983; Heuner et al., 1995; Cazalet et al., 2010; Kozak et al., 2010; Brzuszkiewicz et al., 2013). With the advance in molecular techniques and the ability to produce and to process metagenomics datasets, it was found that some of the non-flagellated *Legionella* have still parts of the flagellar regulon, mainly genes with regulator functions.

However, in *Legionella* flagellum synthesis is associated with the expression of a virulence phenotype; and motility can be seen as a virulence and a fitness factor in *Legionella* and other bacteria. The alternative sigma factor FliA is also involved in the expression of virulence traits. FliA-dependent putative virulence genes were already identified by initial investigations that need to be extended. Also, additional investigations are needed to determine the role of FliA and molecular mechanisms of FliA-FlgM interactions in *Legionellae*. FlgM and FliA, two main players involved in the expression of the flagellum genes, are still present in non-flagellated *Legionella*, a promising take-off for future investigations. Nevertheless, the flagellum is not necessarily needed for an intracellular replication within host cells. Moreover, in some hosts the Naip5/Ipaf-dependent recognition of flagellin can cause an innate immune response leading to resistance against *Legionella* infections (Molofsky et al., 2006; Ren et al., 2006). For example, it was reported that biofilm-derived *L. pneumophila* without flagellin expression evade the innate immune response in macrophages (Abu Khweek et al., 2013), as it was suggested for the non-flagellated *L. longbeachae* (Cazalet et al., 2010; Kozak et al., 2010). It seems that under certain conditions, the loss of the flagellum may increase the fitness of bacteria. For instance, *L. pneumophila* which can be found mainly in aquatic environments, is still flagellated whereas *L. longbeachae* which can be found predominantly in soil, is non-flagellated (Kozak et al., 2010). Nevertheless, if the loss of the flagellar system from *Legionella* species depends on the habitat or environmental conditions remains unanswered.

As outlined, flagellated and non-flagellated *Legionella* are positive for genes belonging to the chemotaxis operon. The ability of *Legionella* to swarm and to show off a chemotaxis behavior has not yet been reported. Interestingly, some chemotaxis-positive and flagellar operon-negative *Legionella* (e.g., *L. longbeachae*) give credit to the assumption that chemotaxis genes may not be involved in flagellum-mediated motility. Recent investigations could even show that chemotaxis sensory systems—different from those found in *E. coli*—in distinct bacteria (e.g., *Myxococcus* spp., *Geobacter* spp.) are not necessarily involved in bacterial flagellum-mediated motility (Kirby, 2009; Kozak et al., 2010). Chemotaxis-like systems seem to be involved among other things in type IV pilus-based motility and cell to cell interaction and/or social motility (Kirby, 2009; Kozak et al., 2010). Accordingly, additional experimentations are needed to investigate the role of the chemotaxis operon in flagellated and non-flagellated, chemotaxis-positive *Legionella*.

## Author contributions

SA and KH contributed substantial to the conception and design of the work. SA and KH wrote the paper.

### Conflict of interest statement

The authors declare that the research was conducted in the absence of any commercial or financial relationships that could be construed as a potential conflict of interest.
